# Decoding ceRNA regulatory network in the pulmonary artery of hypoxia-induced pulmonary hypertension (HPH) rat model

**DOI:** 10.1186/s13578-022-00762-1

**Published:** 2022-03-07

**Authors:** Jun Wang, Yanqin Niu, Lingjie Luo, Zefeng Lu, Qinghua Chen, Shasha Zhang, Qianwen Guo, Li Li, Deming Gou

**Affiliations:** Shenzhen Key Laboratory of Microbial Genetic Engineering, Vascular Disease Research Center, College of Life Sciences and Oceanography, Guangdong Provincial Key Laboratory of Regional Immunity and Disease, Carson International Cancer Center, School of Medicine, Shenzhen University, Shenzhen, 518060 China

**Keywords:** HPH, ceRNA regulatory network, Differentially expressed RNAs, Diagnosis of PAH

## Abstract

**Background:**

Hypoxia-induced pulmonary hypertension (HPH) is a lethal cardiovascular disease with the characteristic of severe remodeling of pulmonary vascular. Although a large number of dysregulated mRNAs, lncRNAs, circRNAs, and miRNAs related to HPH have been identified from extensive studies, the competitive endogenous RNA (ceRNA) regulatory network in the pulmonary artery that responds to hypoxia remains largely unknown.

**Results:**

Transcriptomic profiles in the pulmonary arteries of HPH rats were characterized through high-throughput RNA sequencing in this study. Through relatively strict screening, a set of differentially expressed RNAs (DERNAs) including 19 DEmRNAs, 8 DElncRNAs, 19 DEcircRNAs, and 23 DEmiRNAs were identified between HPH and normal rats. The DEmRNAs were further found to be involved in cell adhesion, axon guidance, PPAR signaling pathway, and calcium signaling pathway, suggesting their crucial role in HPH. Moreover, a hypoxia-induced ceRNA regulatory network in the pulmonary arteries of HPH rats was constructed according to the ceRNA hypothesis. More specifically, the ceRNA network was composed of 10 miRNAs as hub nodes, which might be sponged by 6 circRNAs and 7 lncRNAs, and directed the expression of 18 downstream target genes that might play important role in the progression of HPH. The expression patterns of selected DERNAs in the ceRNA network were then validated to be consistent with sequencing results in another three independent batches of HPH and normal control rats. The diagnostic effectiveness of several hub mRNAs in ceRNA network was further evaluated through investigating their expression profiles in patients with pulmonary artery hypertension (PAH) recorded in the Gene Expression Omnibus (GEO) dataset GSE117261. Dysregulated POSTN, LTBP2, SPP1, and LSAMP were observed in both the pulmonary arteries of HPH rats and lung tissues of PAH patients.

**Conclusions:**

A ceRNA regulatory network in the pulmonary arteries of HPH rats was constructed, 10 hub miRNAs and their corresponding interacting lncRNAs, circRNAs, and mRNAs were identified. The expression patterns of selected DERNAs were further validated to be consistent with the sequencing result. POSTN, LTBP2, SPP1, and LSAMP were suggested to be potential diagnostic biomarkers and therapeutic targets for PAH.

**Supplementary Information:**

The online version contains supplementary material available at 10.1186/s13578-022-00762-1.

## Background

Chronic hypoxia-induced pulmonary hypertension (HPH) is one of the most devastating cardiovascular diseases that is characterized by remodeling of pulmonary vascular and persistent elevation of pulmonary arterial pressure [1]. Increased right heart load is another main characteristic of HPH, which may lead to disturbance of the pulmonary circulatory, right heart failure, and ultimately death [2]. Although a great breakthrough has been made in illuminating the pathogenesis, identifying prognostic biomarkers, and improving therapeutic strategies of HPH, the overall incidence and mortality rates remain high [3, 4]. Therefore, unveiling novel insights into the mechanisms involved in the development of HPH is of great significance in facilitating further understanding of HPH.

A large number of RNAs participating in HPH development have been characterized in several previous studies [5, 6]. The RNA-mediated regulatory networks consisting of both coding mRNAs and noncoding RNAs (lncRNAs, circRNAs, and miRNAs) play an important role in the progression of HPH. Dysregulated mRNAs that contributed to the remodelling of pulmonary artery, which is mainly due to excessive proliferation and migration of pulmonary artery smooth muscle cells (PASMCs), have been extensively reported [7–13]. These mRNAs were implicated in TGF-β signaling [7], Notch signaling [9], PI3K/AKT/mTOR signaling [8], PPAR signaling pathways [12, 13], and so on. Recent studies have revealed that noncoding RNAs including lncRNAs, circRNAs, and miRNAs were essential in mediating HPH pathogenesis as well [14–23]. For instance, lncRNA-MEG3 was proved to be upregulated in the cytoplasm of hypoxic PASMCs, which degraded the cytoplasmic miR-328-3p, and subsequently led to the upregulation of insulin-like growth factor receptor (IGF1R). LncRNA-MEG3 was ultimately demonstrated to be a novel biomarker and therapeutic target of HPH [24]. In addition, miR-483 [18], miR-182-3p [19], miR-125-5p [20], circRNA CDR1as [22], hsa_circ_0016070 [23] and circ-calm4 [18] were identified to function through RNA-RNA interactions in mediating the pathogenesis of HPH or pulmonary artery hypertension (PAH). Nevertheless, the overall RNA interacting networks at the transcriptomic level in the pulmonary arteries of HPH rats remain elusive.

Moreover, several RNAs, including lncRNA, circRNA, and other RNAs were recently found to interact with each other and act as natural miRNA sponges to form competing endogenous RNA (ceRNA) networks that participate in the regulation of many biological processes [25, 26]. However, the role of the ceRNA networks in regulating the remodeling of the pulmonary artery during HPH development has not been characterized.

**Fig. 1:**
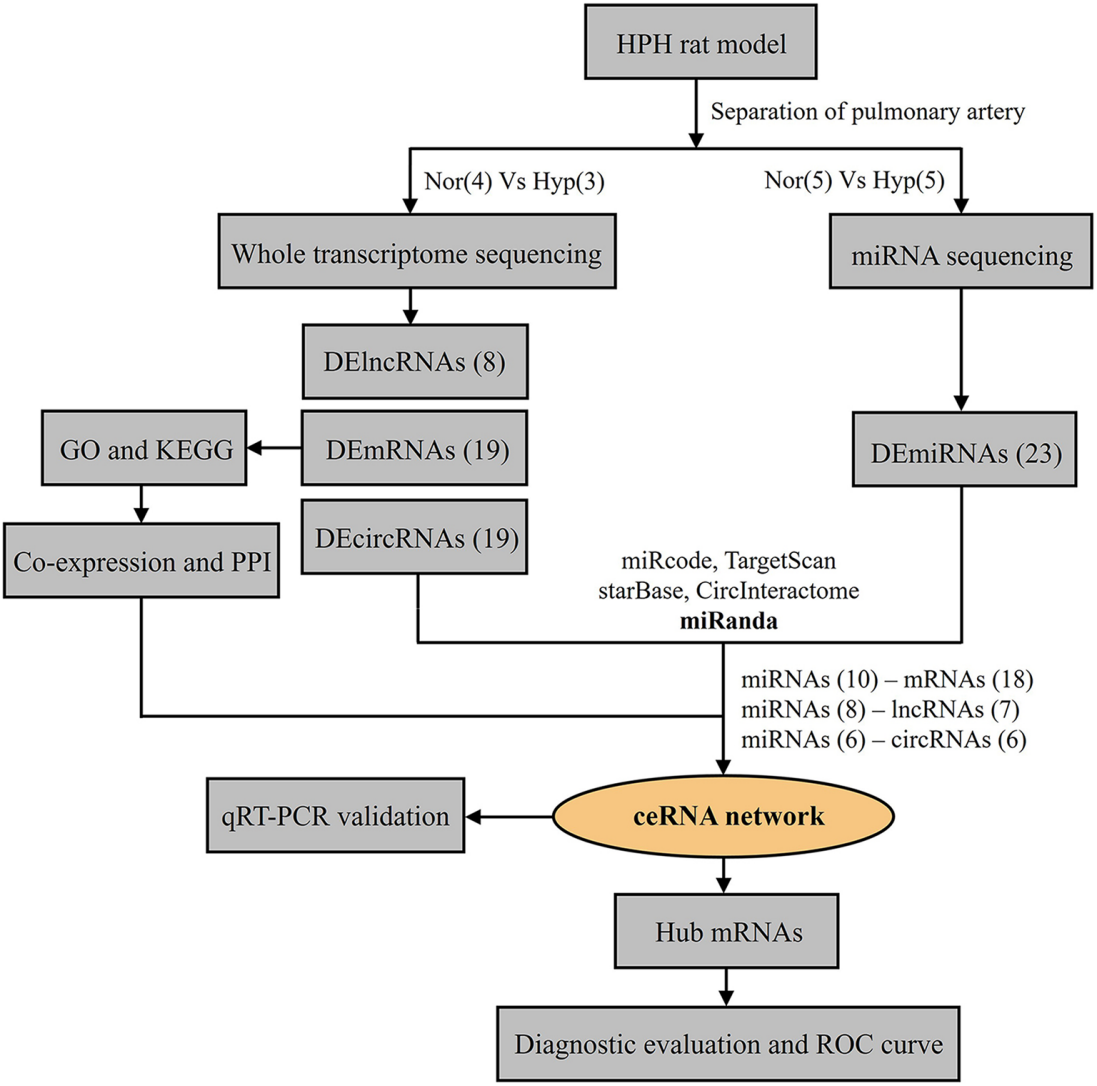
Workflow of the study design. HPH: hypoxia-induced pulmonary hypertension; DElncRNAs: differentially expressed lncRNAs; DEmRNAs: differentially expressed mRNAs; DEcircRNAs: differentially expressed circRNAs; DEmiRNAs: differentially expressed miRNAs; GO: Gene Ontology; KEGG: Kyoto Encyclopedia of Genes and Genomes; PPI: protein–protein interactions; ceRNA: competing endogenous RNA; qRT-PCR: real-time reverse transcription-PCR; ROC: receiver operating characteristic curve

To reveal the ceRNA regulation network during HPH progression, we profiled whole transcriptome (mRNAs, lncRNAs, circRNAs, and miRNAs) in pulmonary arteries of HPH rats as well as the normal controls. According to the workflow in this study (Fig. 1), differentially expressed miRNAs (DEmiRNAs) were identified as potential hub genes for constructing a ceRNA regulatory network through predicting their interacting relationships with other differentially expressed RNAs (DERNAs). Furthermore, functional enrichment and protein–protein interaction (PPI) analysis were conducted to identify the hub proteins and elucidate possible regulatory mechanisms in HPH development. DERNAs involved in the ceRNA regulatory network were then validated through real-time reverse transcription-PCR (qRT-PCR). Potential hub mRNAs were finally evaluated for their diagnostic effectiveness in patients with PAH. Our study, for the first time, revealed the ceRNA regulatory network that presented in the pulmonary artery during HPH development, and identified potentially dysregulated mRNAs for PAH diagnosis. We hope the results from this study may pave the way for the discovery of novel diagnostic biomarkers and therapeutic targets of HPH.

## Results

### Construction of HPH rat model

**Fig. 2:**
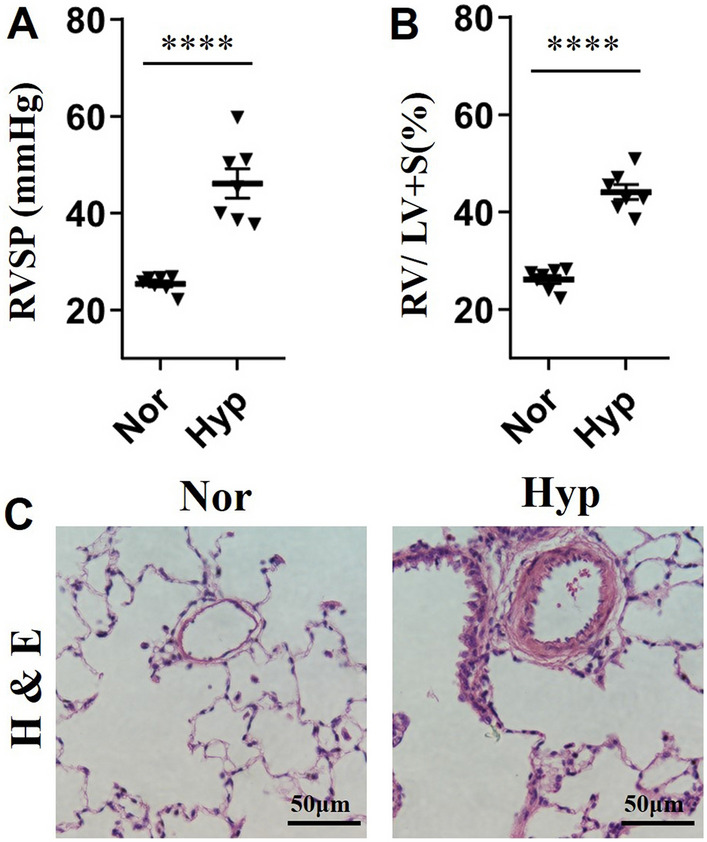
Construction of hypoxia-induced pulmonary hypertension (HPH) rat model. **A** The recorded right ventricular pressure (RVSP) of the first batch of HPH rats and normal controls. **B** The calculated right ventricular hypertrophy index (RVHI) of the first batch of HPH rats and normal controls. **C** Haematoxylin and eosin (H&E) staining of pulmonary arteries from HPH rats and normal controls. Nor: normal control rats; Hyp: HPH rats. ****indicates *p* < 0.0001

To construct the HPH rat model, healthy rats were exposed to chronic hypoxia, body weight, right ventricular pressure (RVSP), and right ventricular hypertrophy index (RVHI) of the rats were evaluated at 21 days post hypoxia. Four batches of HPH rats were constructed in this study, the first batch of HPH rats was used for high throughput RNA (mRNAs, lncRNAs, circRNAs, and miRNAs) sequencing, whereas the other three batches of HPH rats was utilized for qRT-PCR validation. RVSP and RVHI were significantly increased in the first batch of HPH rats compared with the control rats (Fig. 2A, B). Moreover, obvious pulmonary vascular remodeling in HPH rats was confirmed by hematoxylin and eosin (H&E) staining and increased wall thickness of pulmonary artery (Fig. 2C). Similar induction of the HPH rats was also observed for the other three batches of rats (Additional file 1: Table S1).

### Identification of differentially expressed RNAs (DERNAs)

Pulmonary arteries from both HPH and normal rats were separated from connective tissues and cleaned for total RNA isolation, which was then subjected to whole transcriptome sequencing and miRNA sequencing respectively.

**Fig. 3:**
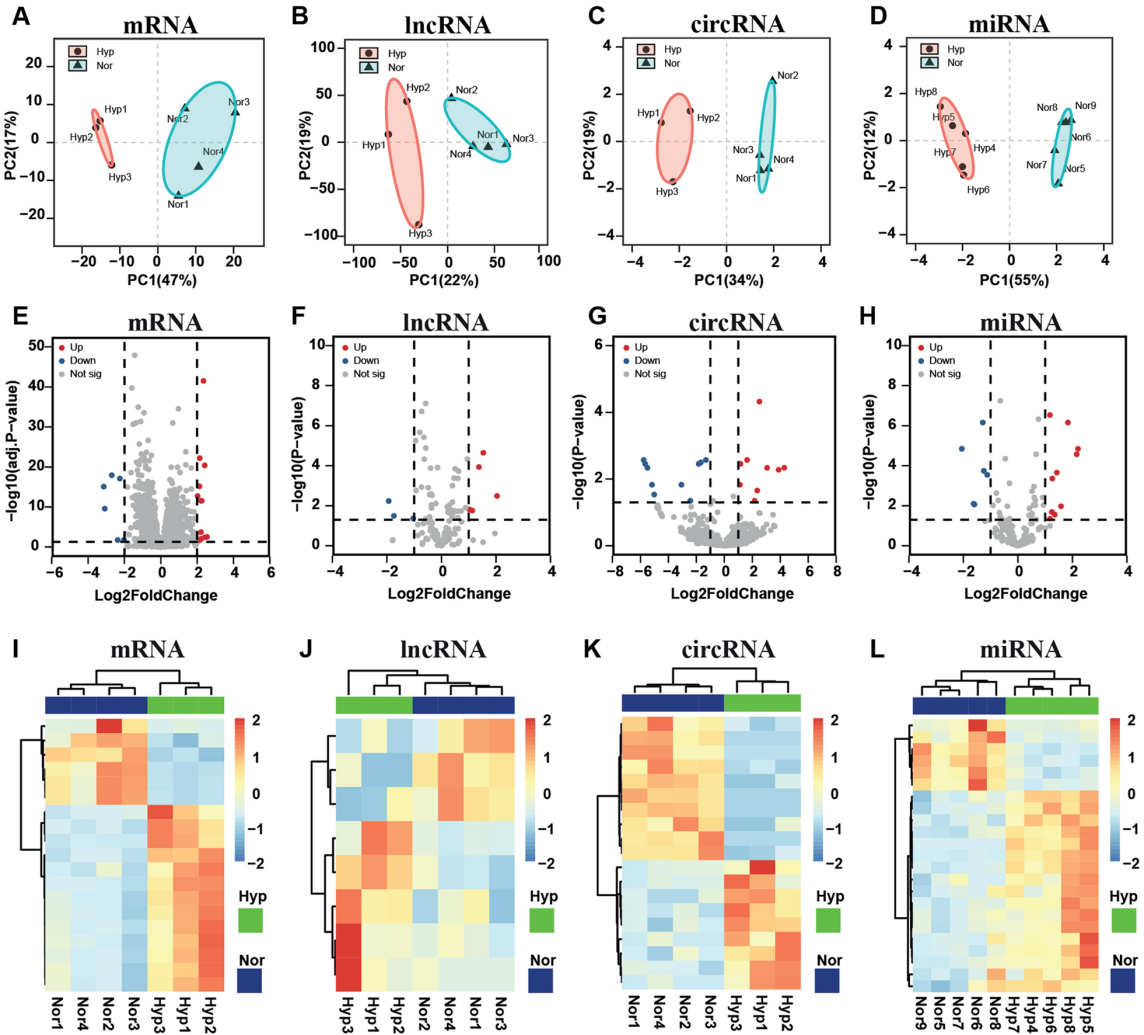
Identification of differentially expressed RNAs (DERNAs). Principal component analysis (PCA) of replicates from both hypoxia-induced pulmonary hypertension (HPH) (red) and control (blue) samples. Samples were clustered according to the expression of 500 most variable mRNAs (**A**), lncRNAs (**B**), circRNAs (**C**), and miRNAs (**D**) in the sequencing dataset. Ellipses represent 95% confidence intervals for the groups. The volcano plot of DEmRNAs (**E**), DElncRNAs (**F**), DEcircRNAs (**G**), and DEmiRNAs (**H**) between HPH and control samples. Red and blue dots represent downregulated and upregulated DERNAs in HPH rats respectively. The horizontal line represents the value of the *padj* < 0.05 (**E**) or *p* < 0.05 (**F**–**H**); the vertical dotted line represents the value of |Log2FoldChange| > 2 (**E**) or |Log2FoldChange| > 1 (**F**–**H**). Expression heatmap of DEmRNAs (**I**), DElncRNAs (**J**), DEcircRNAs (**K**), and DEmiRNAs (**L**) between HPH and control samples. Unsupervised hierarchical clustering analysis of the DERNAs was performed. Orange color indicates higher expression; blue color indicates lower expression. Nor: normal control rats; Hyp: HPH rats

Clean reads were generated through quality control of the raw sequencing reads and were then mapped to the primary assembly of rat genome (RGSC 6.0), and mature rat miRNA sequences listed in miRbase (www.mirbase.org, release 22) (Additional file 1: Table S2). To assess the accuracy and reliability of sequencing result, principal component analysis (PCA) was conducted to analyze the expression profiles of all identified RNAs (mRNAs, lncRNAs, circRNAs, and miRNAs), clear separation between hypoxic and normal rats was observed (Fig. 3A–D), suggesting the applicability of the data for further analysis.

To eliminate those inconsistencies and variations among different rats, a strict criterion was set to identify DERNAs between HPH and control rats in this study. In general, we first filtered the relatively low expressed RNAs by dropping those RNAs with median fragments per kilobase per million mapped reads (FPKM) or transcript per million (TPM) value less than 10 for mRNAs (FPKM), 5 for lncRNAs (FPKM), circRNAs (TPM) and miRNAs (TPM) among all tested samples. Then screening for DERNAs (|Log2FoldChange| > 2 and *padj* < 0.05 for mRNAs; |Log2FoldChange| > 1 and *p* < 0.05 for lncRNAs, circRNAs, and miRNAs) were performed, 19 significant DEmRNAs (13 up- and 6 downregulated), 8 significant DElncRNAs (5 up- and 3 downregulated), 19 significant DEcircRNAs (9 up- and 10 downregulated) and 23 DEmiRNAs (17 up- and 6 downregulated) were eventually identified in the pulmonary arteries of the HPH rats compared with the control rats. The volcano plot suggested the significant statistical differences relative to the magnitude of differences for every single gene between HPH and control groups (Fig. 3E–H). In addition, heatmap of the significant dysregulated RNAs showed hierarchical clustering between HPH and normal control rats (Fig. 3I–L).

### Gene ontology and KEGG pathway analysis

**Fig. 4:**
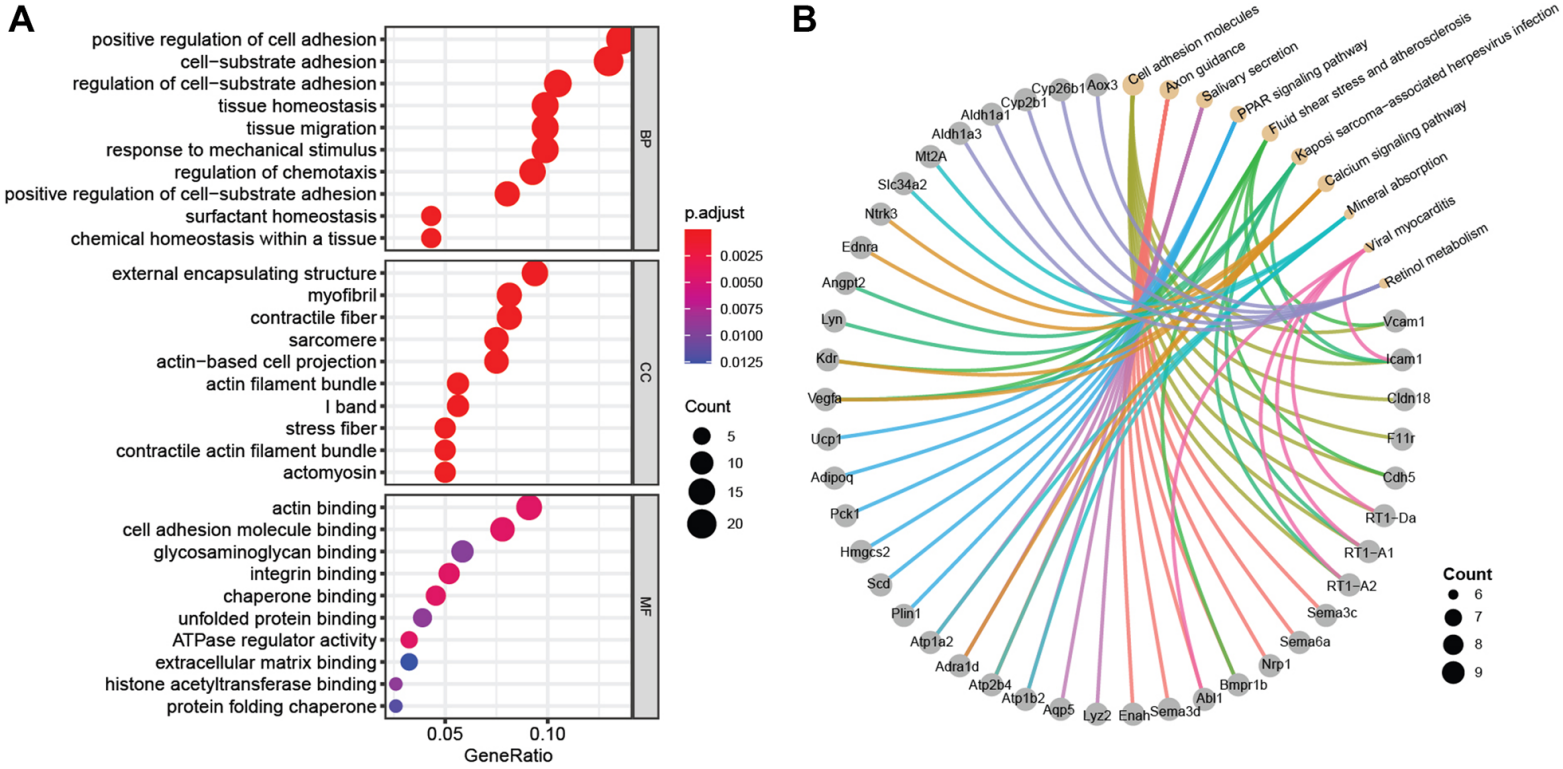
Functional classifications and pathway enrichment analysis of DERNAs. **A** Gene Ontology (GO) analysis of DEmRNAs between HPH and normal samples. Three aspects including biological process (BP), cellular component (CC), and molecular function (MF) were analyzed. **B** Kyoto Encyclopedia of Genes and Genomes (KEGG) pathway analysis of DEmRNAs between HPH and normal samples. Yellow dots indicate the top 10 enriched pathways; grey dots indicate the genes involved in the corresponding pathways

To further characterize the regulatory network in the pulmonary artery post hypoxia, Gene Ontology (GO) and Kyoto Encyclopedia of Genes and Genomes (KEGG) pathway analysis were conducted for the DEmRNAs (|Log2FoldChange| > 1 and *padj* < 0.05). Top 10 enriched GO terms were mainly associated with positive regulation of cell adhesion (gene ratio = 22/162, *p* = 1.54E−10), cell-substrate adhesion (gene ratio = 21/162, *p* = 2.04E−11), external encapsulating structure (gene ratio = 15/160, *p* = 2.87E−06), myofibril (gene ratio = 13/160, *p* = 1.40E−07), actin binding (gene ratio = 14/154, *p* = 3.67E−05), cell adhesion molecule binding (gene ratio = 12/154, *p* = 1.26E−05), and so on (Fig. 4A; Additional file 1: Table S3).

Moreover, top 10 KEGG pathways (*p* < 0.05) with the highest gene ratio were also identified (Fig. 4B), including cell adhesion molecules (gene ratio = 8/90, *p* = 0.00034), axon guidance (gene ratio = 7/90, *p* = 0.00236), salivary secretion (gene ratio = 7/90, *p* = 0.00016), PPAR signaling pathway (gene ratio = 6/90, *p* = 0.00022), fluid shear stress and atherosclerosis (gene ratio = 6/90, *p* = 0.00424), kaposi sarcoma-associated herpesvirus infection (gene ratio = 6/90, *p* = 0.02227), calcium signaling pathway (gene ratio = 6/90, *p* = 0.033673612), mineral absorption (gene ratio = 5/90, *p* = 0.00024), viral myocarditis (gene ratio = 5/90, *p* = 0.00167), retinol metabolism (gene ratio = 5/90, *p* = 0.00176), and so on (Additional file 1: Table S4). As the enriched pathways were usually present in proliferation or metastasis of cancer cells, these results further suggested the cancer-like pathobiology in the pulmonary arteries of HPH rats.

### Construction of a potential lncRNA/circRNA-miRNA-mRNA ceRNA regulatory network

**Fig. 5:**
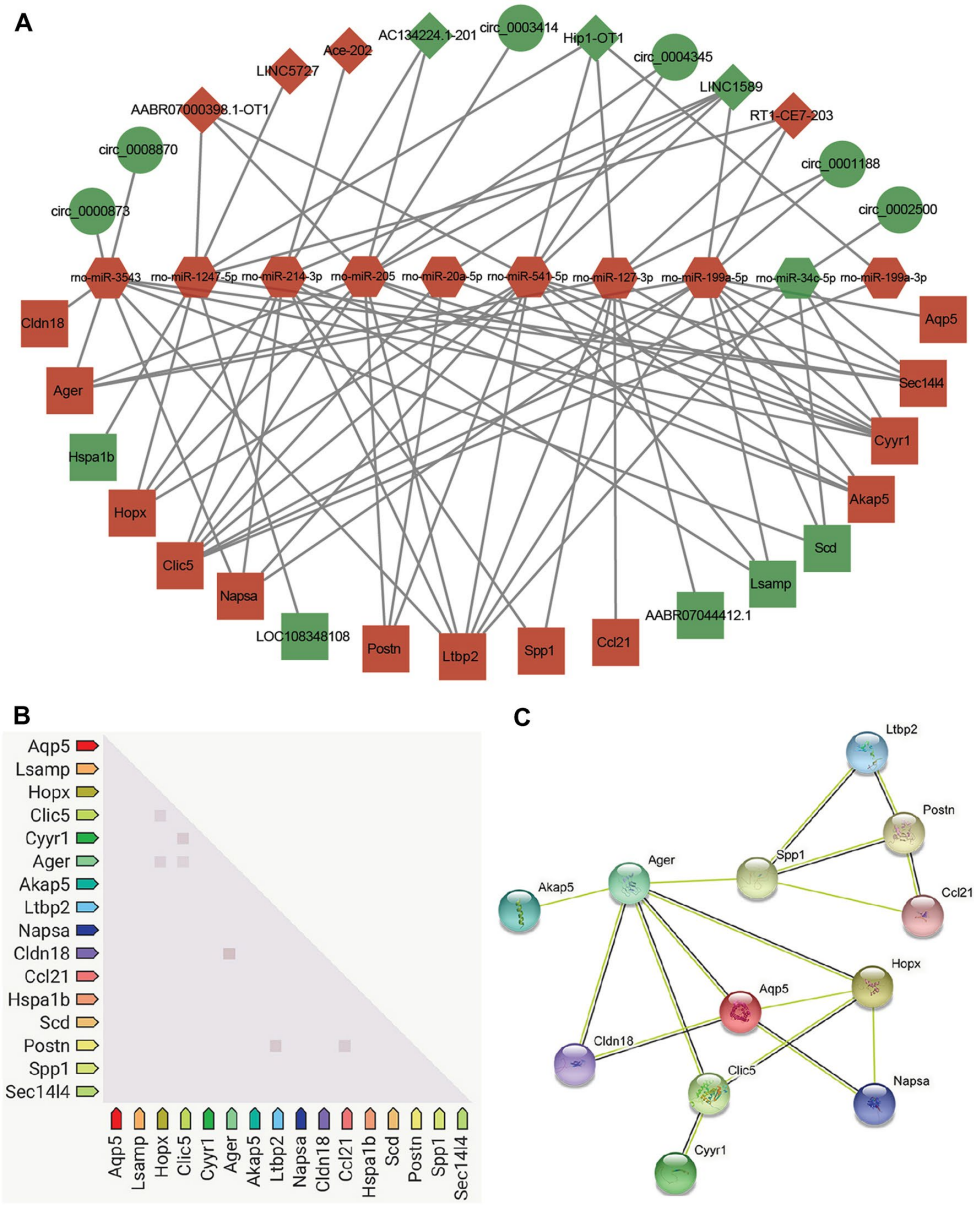
Potential competing endogenous RNA (ceRNA) regulatory network and protein–protein interactions (PPI) analysis in the pulmonary artery of HPH rats. **A** LncRNA/circRNA-miRNA-mRNA ceRNA regulatory network constructed in this study. The ceRNA regulatory network includes 10 miRNAs, 6 circRNAs, 7 lncRNAs, and 18 mRNAs. Red color indicates upregulated, green color indicates downregulated; circles indicate circRNAs, retangles indicate mRNAs, diamonds indicate lncRNAs, hexagons indicate miRNAs. **B** Co-expression analysis of DEmRNAs by STRING database. The squares represent gene association, more intense color of the squares represent higher association score. **C** Results of PPI analysis of DEmRNAs by STRING database. The balls represent the gene nodes, the connecting lines represent the interactions between genes and figures insides the balls represent protein structure

According to the ceRNA hypothesis, lncRNAs could compete with circRNAs for the same miRNAs and further impact downstream gene expression. To obtain the competing relationships, we predicted the interacting possibilities between DElncRNAs–DEmiRNAs, DEcircRNAs–DEmiRNAs, and DEmiRNAs–DEmRNAs. We found that a total of 10 DEmiRNAs (9 up- and 1 downregulated) could be targeted by 7 DElncRNAs (4 up- and 3 downregulated) and 6 DEcircRNAs (6 downregulated). Furthermore, these DEmiRNAs could target 18 DEmRNAs (13 up- and 5 downregulated) (Table 1). Finally, an lncRNA/circRNA-miRNA-mRNA ceRNA regulatory network that responds in the pulmonary arteries of HPH rats was constructed based on the interacting relationships (Fig. 5A), which was composed of 41 nodes and 86 connections.

**Table 1:** The DERNA interacting relationships in the ceRNA regulatory network

DEmiRNAs	DEmRNAs	DElncRNAs	DEcircRNAs
rno-miR-1247-5p	Hopx,Sec14l4, LOC108348108,Hspa1b	AABR07000398.1-OT1, LINC5727, Hip1-OT1, RT1-CE7-203	N.A
rno-miR-127-3p	Ccl21, Ltbp2, Cyyr1, Spp1, Ager, AABR07044412.1	Hip1-OT1, RT1-CE7-203	circ_0001188
rno-miR-199a-3p	Clic5	Hip1-OT1	N.A
rno-miR-199a-5p	Napsa, Scd, Akap5, Ltbp2, Aqp5, Clic5, Cyyr1, Lsamp, Sec1414, Ager	LINC1589, RT1-CE7-203	circ_0001188
rno-miR-205	Postn, Akap5, Ltbp2, Clic5, Cyyr1, Hopx, Ager	AABR07000398.1-OT1, AC134224.1–201, LINC1589	circ_0003414, circ_0004345
rno-miR-20a-5p	Postn, Clic5, Cyyr1	LINC1589	N.A
rno-miR-214-3p	Napsa, Akap5, Ltbp2, Clic5, Lsamp, Spp1, Hopx	AC134224.1–201,Ace-202, LINC1589	N.A
rno-miR-34c-5p	Scd, Ltbp2, Clic5, Cyyr1, Sec14l4	N.A	circ_0002500
rno-miR-3543	Napsa, Scd, Ltbp2, Cldn18, Cyyr1, Sec14l4, Ager	N.A	circ_0000873, circ_0008870
rno-miR-541-5p	Napsa, Postn, Akap5, Ltbp2, Clic5, Cyyr1, Lsamp, Hopx, Sec14l4	AABR07000398.1-OT1, LINC1589, Hip1-OT1	circ_0004345

In addition, the co-expression pattern of the DEmRNAs in the ceRNA network was also investigated. We found seven co-expression gene pairs including HopX-Clic5, HopX-Ager, Clic5-Cyy1, Clic5-Ager, Cldn18-Ager, Postn-Ltbp2, and Postn-Ccl21 (Fig. 5B). Furthermore, PPI analysis of the DEmRNAs suggested the hub role of Postn, Spp1, Ager, Aqp5, Clic5, and HopX in mediating the response of pulmonary artery to hypoxia (Fig. 5C).

### Validation of DERNAs in ceRNA network

**Fig. 6:**
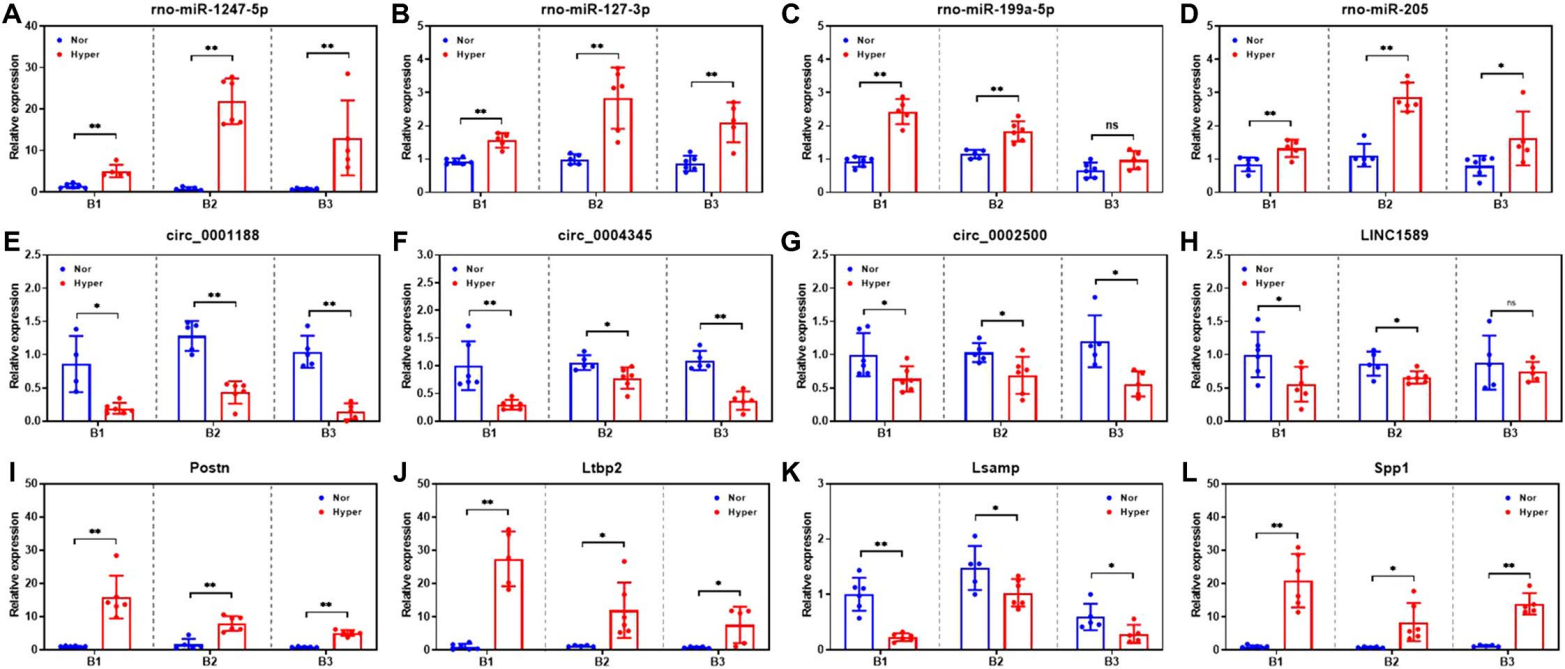
Expression profiles of selected DERNAs in the ceRNA regulatory network. Expression level of rno-miR-1247-5p (**A**), rno-miR-127-3p (**B**), rno-miR-199a-5p (**C**), rno-miR-205 (**D**), circ_0001188 (**E**), circ_0004345 (**F**), circ_0002500 (**G**), LINC1589 (**H**), Postn (**I**), Ltbp2 (**J**), Lsamp (**K**) and Spp1 (**L**) in the pulmonary arteries of three independent batches (B1, B2 and B3) of both HPH and normal rats. *p < 0.05; **p < 0.01; ns, not significant; Nor: normal control rats; Hyp: HPH rats

To validate the potential interaction and expression profiles of DERNAs in the ceRNA network, the expression level of selected DERNAs were verified by qRT-PCR on another three independent batches of HPH and normal control rats. The expression of rno-miR-1247-5p, rno-miR-127-3p, rno-miR-205, Postn, Ltbp2, and Spp1 were demonstrated to be upregulated in all three batches of rats, which was as expected according to the sequencing results (Fig. 6A, B, D, I, J, L). Moreover, circ_0001188, circ_0004345, circ_0002500, and Lsamp were also proved to be downregulated in all three batches of rats, which was consistent with the sequencing result (Fig. 6E–G, K). Whereas rno-miR-199a-5p and LINC1589 showed significant dysregulation in two out of the three batches of rats (Fig. 6C, H). These results further supported the lncRNA/circRNA-miRNA-mRNA regulatory network constructed in this study.

### Evaluation of hub DEmRNAs for diagnostic effectiveness of PAH

**Fig. 7:**
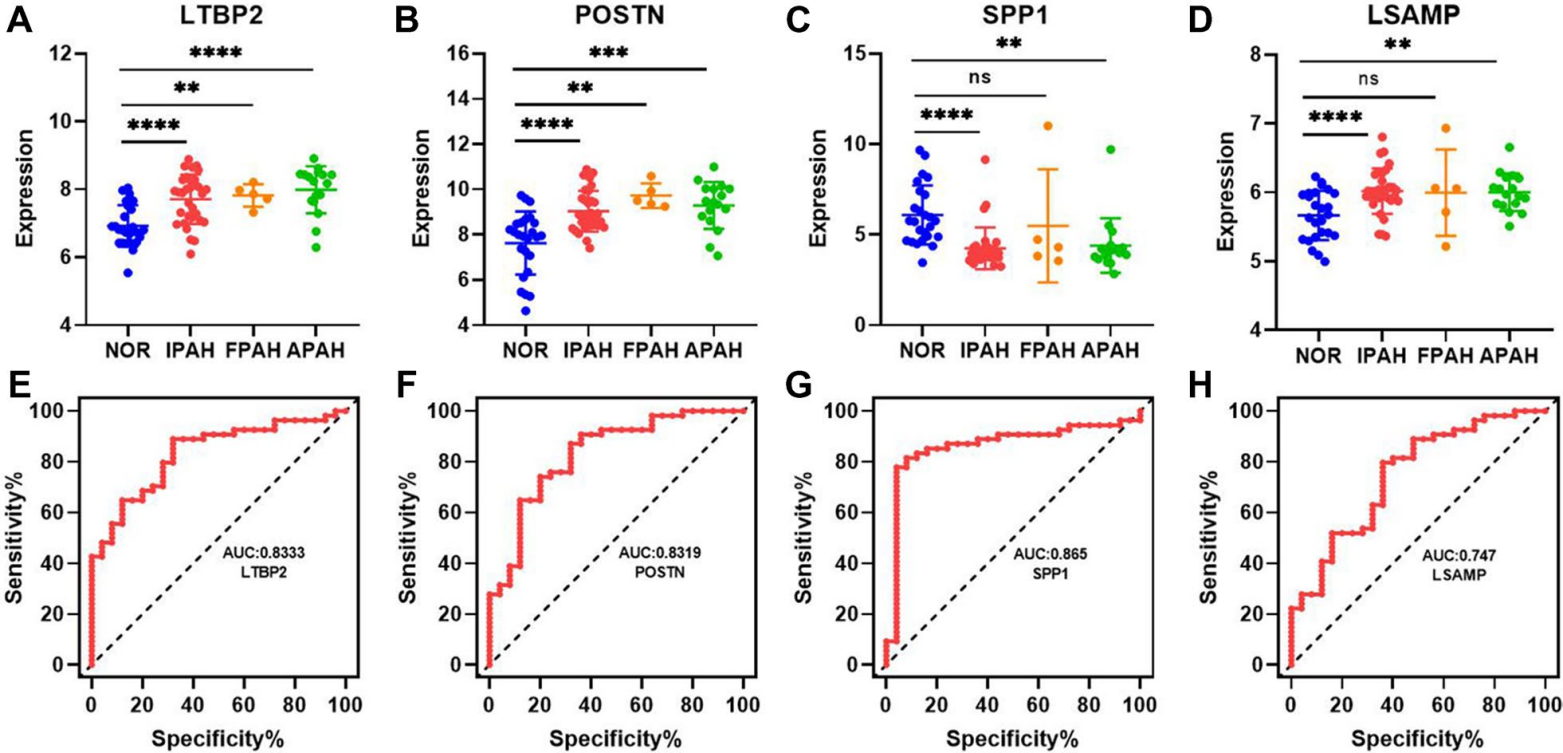
Evaluation of the diagnostic value of potential hub mRNAs in patients with pulmonary artery hypertension (PAH). Expression profiles of LTBP2 (**A**), POSTN (**B**), SPP1 (**C**), and LSAMP (**D**) in the lung tissues of PAH patients and normal individuals. NOR: normal individuals. IPAH: idiopathic PAH; HPAH: heritable PAH, APAH: associated PAH (connective tissue disease, congenital heart defects, anorexigen/stimulant drug use, and so on). *p < 0.05, **p < 0.01, ***p < 0.001, and ****p < 0.0001. ROC curve analysis of potential diagnostic mRNAs. The AUC curve showed the effectiveness of LTBP2 (**E**), POSTN (**F**), SPP1 (**G**), and LSAMP (**H**) for the detection of the occurrence of PAH

To further explore whether the pulmonary artery-associated DEmRNAs in the ceRNA network were associated with clinical diagnosis, the expression of selected hub mRNAs were investigated in the dataset of GSE117261, which recorded gene expression profiles in the lung tissues of 25 normal individuals, 32 patients with idiopathic PAH (IPAH); 5 patients with heritable PAH (HPAH), 17 connective tissue disease, congenital heart defects, anorexigen/stimulant drug use-associated PAH (APAH). Finally, 4 DEmRNAs were found significantly dysregulated in patients with PAH. Higher expression of latent transforming growth factor beta binding protein 2 (LTBP2) and periostin (POSTN) was found in all PAH patients (Fig. 7A, B), whereas lower expression of secreted phosphoprotein 1 (SPP1) and limbic system associated membrane protein (LSAMP) was found in most of the PAH patients except the HPAH patients (Fig. 7C, D). Moreover, the diagnostic value of LTBP2, POSTN, SPP1, and LSAMP in differentiating tissues from PAH patients and healthy individuals was evaluated. LTBP2 and POSTN were found to be upregulated in both pulmonary arteries of HPH rats and lung tissues of PAH patients. Area under the curve (AUC) of 0.8333 (95% confidence interval (CI) 0.7429–0.9237) for LTBP2 and AUC of 0.8319 (95% CI 0.7336–0.9301) for POSTN were identified (Fig. 7E, F). Although SPP1 was found to have an opposite expression pattern in the pulmonary arteries of HPH rats and lung tissues of PAH patients, it exhibited the best diagnostic effectiveness with an AUC of 0.8652 (95% CI 0.7723–0.9580) (Fig. 7G). Similarly, LSAMP was found to have an AUC of 0.747 (95% CI 0.6300–0.8648) in diagnosing PAH (Fig. 7H).

## Discussion

With the increasing incidence and prevalence of HPH reported in the last decade [3, 4], a more intensive understanding of the molecular mechanism during HPH development is urgently required for achieving better diagnosis and therapy. In this study, we constructed a transcriptomic regulatory network based on high throughput RNA sequencing results of the pulmonary arteries from HPH rats. We hope that the ceRNA network identified in this study could provide comprehensive and novel insights into the pathogenesis as well as potential therapeutic targets of HPH.

In this study, only high expressed RNAs were used for differentially expression analysis to eliminate variations presented in HPH rats. DEmRNAs (|Log2FoldChange| > 1 and *padj* < 0.05) identified in the pulmonary arteries were proved to participate in cell adhesion, axon guidance, PPAR signaling pathway, and calcium signaling pathway after hypoxia. In accordance with our findings, DEmRNAs such as Vegfa [27], Ager [28], Ltbp2 [29], Postn [30], Atp2b4 [31], and Ccl21 [32] have been previously reported dysregulated during HPH or PAH development. Although lncRNAs and circRNAs were found to have much lower expression levels compared with mRNAs, 8 novel DElncRNAs and 19 novel DEcircRNAs that responded to hypoxia in the pulmonary arteries were also observed. In addition, among the 23 DEmiRNAs, miR-20a-5p [33], miR-199a-5p [34], miR-34c-5p [35], and miR-214-3p [36] have been reported to be involved in the process of pulmonary vascular remodeling. Profiling the expression of these DERNAs indicated that significant alterations of RNA expression were present in the pulmonary artery upon hypoxia, which might contribute to the pathophysiology of HPH.

Growing evidence suggested that lncRNAs and circRNAs with miRNA binding sites (MREs) could compete with mRNAs for binding to miRNAs, thereby regulating the RNA expression and affecting disease progression. Despite several ceRNA networks and lncRNA-miRNA interactions have been reported in the lung tissue of HPH [37–39], the crosstalk of lncRNA/circRNA-miRNA-mRNA in the pulmonary arteries of HPH rats has never been investigated.

Upon obtaining the DERNAs in the pulmonary arteries of HPH rats, DEmiRNAs were selected as hub nodes for predicting the interacting relationships between DEmiRNAs–DElncRNAs, DEmiRNAs–DEcircRNAs, and DEmiRNAs–DEmRNAs. To eliminate the false positive, a relatively strict threshold was set to screen for the RNA–RNA interactions. Ten miRNAs were finally identified as hub nodes to compete with 7 lncRNAs and 6 circRNAs for directing the expression of 18 mRNAs.

miR-214-3p has been demonstrated to significantly upregulated and mediated the proliferation and migration of PASMCs upon hypoxia by directly targeting ARHGEF12 [36]. In this study, we further extended the potential regulating axis by introducing 2 lncRNAs that might specifically sponge miR-214-3p to regulate the expression of 6 downstream mRNAs. Moreover, miR-199a-3p has been found to directly target Clic5 and promote the cell cycle for cardiomyocyte proliferation and regeneration [40, 41]. A similar regulation axis might also present in the pulmonary artery as several miRNAs including miR-199a-3p were supposed to control the expression of Clic5. Interestingly, another lncRNA Hip1-OT1 was predicted to simultaneously sponge miR-541-5p and miR-199a-3p to affect the expression of Clic5. In addition, downregulated miR-34c-5p was also found to regulate the Clic5 expression, and the regulatory axis might consist of another novel circular RNA circ_0002500.

With emerging evidence showing the critical role of circRNAs in diverse physiological processes, the biological function and molecular diagnostic value of circRNAs in HPH are attracting scientific attention. circRNA CDR1as was recently demonstrated to upregulate calcium/calmodulin-dependent kinase II-delta (CAMK2D) and calponin 3 (CNN3) through sponging miR-7-5p in PASMCs to promote its calcification [22]. Moreover, the hsa_circ_0016070/miR-942/CCND1 regulatory axis was also identified to be associated with HPH through promoting PASMCs proliferation [23]. According to the research result above, we speculated that the DEcircRNAs identified in this study might function synergistically with other DERNAs in the pathogenesis of HPH. According to our hypothesis, both circ_0000873 and circ_0008870 could interact with miR-3543 and thus upregulate downstream mRNAs including Cldn18, Ager, Napsa, and Ltbp2, which were considered to participate in inflammatory and regulation of cell proliferation. Furthermore, circ_0003414/circ_0004345-miR-205, circ_0004345-miR-541-5p, circ_0001188-miR-127-3p, circ_0001188-miR-199a-5p, and circ_0002500-miR-34c-5p were predicted to be circRNA-miRNA regulatory pairs, which together with the downstream mRNAs might cooperatively or independently participate as regulatory axis in HPH development. Nevertheless, the biological functions and the regulatory mechanisms required further clarification.

GO and KEGG analysis performed in this study focused on the DEmRNAs in the pulmonary arteries of HPH rats. The enriched functions and processes include cell adhesion, cell-substrate adhesion, tissue migration, actin binding, glycosaminoglycan binding, extracellular matrix binding, and so on. In parallel with GO analysis, KEGG analysis identified cell adhesion molecules, axon guidance, PPAR signaling pathway, calcium signaling pathway, and so on. These findings were consistent with the fact that the pulmonary vascular remodeling was mainly due to the proliferation and migration of PASMCs.

PPI analysis via STRING database suggested the key role of Ager, Spp1, Clic5, Aqp5, Postn, Ltbp2, and Hopx in the pulmonary artery post hypoxia, which might also be potential diagnostic biomarkers and therapeutic targets for HPH. Co-expression of Hopx-Clic5-Ager, Postn-Ltbp2, and Postn-Ccl21 were further identified, suggesting their synergistic function in the pulmonary artery during hypoxia. Postn, an extracellular matrix encoding protein that involved in tissue remodeling in response to injury, was found to be upregulated in the pulmonary arteries of HPH patients [30]. Accumulated POSTN in the nucleus of the endothelial cells upon hypoxia leads to its dysfunction, whereas extracellular POSTN secreted from the cytoplasm promotes the proliferation and migration of PASMCs and thus lead to the progression of HPH [30]. Moreover, Postn expression was also reported to increase in RV of monocrotaline (MCT)-induced PAH rats, and increased POSTN could further enhance inducible nitric oxide synthase (iNOS) expression and subsequent nitric oxide (NO) production in right ventricular fibroblast (RVFbs) [42]. Since extracellular matrix remodeling is the key phenomenon in cancer cell invasion and metastasis, the remodeling of pulmonary artery initiated by dysregulated Postn further confirmed the cancer-like pathobiology of PAH. In this study, Postn was predicted to be targeted by miR-205, miR-20a-5p, and miR-541, which were speculated to compete with several lncRNAs and circRNAs for binding to Postn. Therefore, the lncRNA/circRNA-miRNA-Postn regulatory axis might be present during the development of HPH and required validation and exploration in the future.

Similarly, expression of Ager was not only found to increase in both human and mouse PASMCs post hypoxia but also significantly upregulated in the pulmonary arteries of hypoxia plus SU5416 (HySU)-induced PAH mice [28]. Activation of Ager could facilitate the extracellular matrix (ECM) deposition and disease progression in HPH [28]. The ceRNA network related to Ager identified in this study was composed of 4 miRNAs, 5 lncRNAs, and 5 circRNAs. Furthermore, Ltbp2 was found to have a diagnostic value for PAH with an AUC of 0.8333 (95% CI 0.7429–0.9237) in this study. Ltbp2 has been demonstrated to be secreted from lung myofibroblasts and could serve as a biomarker for idiopathic pulmonary fibrosis (IPF) [29]. The circEPSTI1/mir-942-5p/LTBP2 regulatory axis was also identified to affect the proliferation and invasion of oral squamous cell carcinoma (OSCC) cells through the acceleration of epithelial-mesenchymal transition (EMT) and phosphorylation of PI3K/Akt/mTOR signaling pathway [43]. Results from these studies further expanded the possibility of Ltbp2 as a diagnostic marker and therapeutic target for PAH. Since one node in the ceRNA network might be involved in multiple regulatory axes, the complex regulatory relationships should be carefully considered and validated.

Nevertheless, the limitations of this study should also be taken into consideration. First, the sample size used for identifying DERNAs was relatively small and thus might lead to increased variations. Second, the RNA–RNA interaction relationships in the ceRNA network were based on a prediction algorithm that required further experimental validation.

In conclusion, a ceRNA regulatory network in the pulmonary artery of HPH rats was constructed, 10 hub miRNAs and their corresponding interacting lncRNAs, circRNAs, and mRNAs were identified. The expression profiles of several RNAs involved in the ceRNA network were validated by qRT-PCR. The diagnostic effectiveness of several hub mRNAs was evaluated.

## Materials and methods

### Construction of HPH rats and sample collection

Healthy male Sprague–Dawley (SD) rats (8-week-old) were randomly divided into normoxia and hypoxia groups with 5 or 6 rats in each group. Rats were exposed to normoxic (21% O2) or hypoxic (10% O2) conditions for 3 weeks respectively. Oxygen concentrations were monitored by detecting probes inside the chambers.

To measure RVSP, rats were initially anesthetized, and the right jugular vein was surgically exposed, then a polyethylene catheter connected to AP-621G (Nihon Kohden, Japan) was finally inserted in the right ventricle (RV) for recoding the RVSP by utilizing MP150 system and AcqKnowledge® 4.2.0 software package (BIOPAC Systems, USA).

After hemodynamic measurement, animals were sacrificed and the chest was opened. The lung, heart, and pulmonary arteries were harvested and washed in clean saline solution at least three times to remove the blood as clean as possible. The pulmonary artery was separated from one lobe of the lung and immediately frozen in liquid nitrogen for RNA isolation. Another lobe of the lung was fixed in formalin to prepare paraffin-embedded tissues for H&E staining. To measure RVHI, the RV was separated from the left ventricle (LV) and the ventricular septum (S). The RVHI was calculated as the ratio of RV weight to the LV plus S weight.

### Whole transcriptome sequencing

Total RNA from pulmonary arteries was isolated with RNAiso Plus (Takara, Japan) and dissolved in RNase-free water according to the instructions provided by the manufacturer. Quality control was conducted for the total RNA by measuring the concentration of RNA by the NanoDrop 2000c Spectrophotometer (Thermo Fisher Scientific, USA), detecting the DNA contamination by gel electrophoresis system EPS 601 (GE Healthcare, USA), evaluating the RNA integrity by Agilent 2100 bioanalyzer (Agilent, USA).

Library construction and sequencing for characterizing mRNA, lncRNA, and circRNA expression were carried out by Novogene Biotechnology Corporation (Beijing, China). In general, sequencing libraries were constructed with 5 μg qualified total RNA as input material. Ribosomal RNA was removed from total RNA, and then the rRNA-depleted RNA was fragmented to 200–300 base pairs (bps). First strand cDNA was synthesized using random hexamer primers and Moloney Murine Leukemia (M-MuLV) reverse transcriptase (RNaseH-), and second strand cDNA synthesis was subsequently performed using DNA polymerase I and RNase H in the reaction buffer with dUTP instead of dTTP. End repair, dA-tailing, adaptor ligation, and size-selection were performed for the double strand cDNA, then library amplification was conducted following USER enzyme treatment, which was subjected to purification. The quality of the library was finally assessed by the Agilent Bioanalyzer 2100 system (Agilent, USA).

Library construction and sequencing for characterizing miRNA expression were carried out following the S-Poly (T) method described in our previous study with some modifications [44]. In general, the sequencing library was constructed from starting material of 500 ng qualified total RNA. One-step poly-adenylation and reverse transcription (Poly(A)/RT) was performed with 5 μl of 4 × reaction buffer, 1 μl 2.5 μlM RT primer, and 1 μl Poly(A)/RT enzyme, the reaction was incubated at 37 °C for 30 min, which was similar to the method described before [44]. Then the exonuclease I (New England Biolabs, USA) was used to eliminate the remaining RT primers. Frist strand cDNA was then ligated to a splint adapter with a random single-stranded overhang and ligation blocking modification according to the method reported previously [45]. Amplification was then executed for the cDNA to generate miRNA sequencing library, which was subjected to purification by AMpure XP beads (Beckman, USA) to select DNA fragments with an averaged size of approximately 180 to 200 bps. The quality of the library was finally assessed by the Agilent Bioanalyzer 2100 system (Agilent, USA).

### Raw sequencing data treatment

Clean reads were obtained by removing raw reads containing adapter and poly-N sequences using in-house python scripts. In addition, low quality reads were eliminated as well. Mapping of the clean reads to rat genome, transcriptome, and mature miRNA sequences from miRbase was performed by using Hisat2 (v2.0.5) or bowtie2 (v2.0.6) [46]. For characterizing transcripts of mRNA, lncRNA, and circRNA, the mapped reads from each sample were assembled by StringTie (v1.3.3) in a reference-based manner. The following principles were used to identify novel lncRNAs: (1) more than 2 exons were found in the transcript; (2) the length of the transcript was larger than 200 bp; (3) coding potential of the transcript was found by CNCI (Coding-Non-Coding-Index) (v2), CPC (Coding Potential Calculator) (cpc-0.9-r2) and PFAM (Pfam Scan) (v1.3) simultaneously. Furthermore, overlapping circRNAs identified by both find_circ and CIRI (V2.0.5) from each sample were considered to be novel circRNAs. Reads mapped to mRNA, lncRNA and circRNA were counted by StringTie (v1.3.3). For characterizing miRNA expression, reads mapped to mature miRNA sequence were counted by an in-house python script. The expression of mRNA, lncRNA, circRNA, and miRNA was calculated using FPKM and TPM methods respectively.

### Identification of DERNAs

Median FPKM or TPM value among all tested samples was calculated for each mRNA, lncRNA, circRNA, and miRNA. Threshold of 10 for mRNAs, 5 for lncRNAs, circRNAs, and miRNAs were used to eliminate low expressed RNAs. After selecting the pre-treated data, DEmRNAs (|Log2FoldChange| > 2 and *padj* < 0.05), DElncRNAs (|Log2FoldChange| > 1 and *p* < 0.05), DEcircRNAs (|Log2FoldChange| > 1 and *p* < 0.05) and DEmiRNAs (|Log2FoldChange| > 1 and *p* < 0.05) were determined by DEseq2 (v1.32.0) R package. DERNAs were then illustrated in volcano and heatmap by ggplot2 (v3.3.3) and pheatmap (v1.0.12) R packages.

### Gene function annotation

GO analysis was conducted based on DEmRNAs (|Log2FoldChange| > 1 and *padj* < 0.05) to evaluate enrichment for biological processes (BP), cellular component (CC), and molecular function (MF) annotations with clusterProfiler (v4.0.0) R package. KEGG analysis was also performed to enrich the signaling pathways associated with DEmRNAs (|Log2FoldChange| > 1 and *padj* < 0.05) using clusterProfiler (v4.0.0) R package. GO terms and KEGG pathways with enriched genes ≥ 2 and *p* < 0.05 were selected for further analysis. The top 10 ranked GO terms and KEGG pathways containing most genes were visualized by ggplot2 (v3.3.3) R package.

### Prediction of targeting relationship

RNA regulatory network among 19 DEmRNAs, 8 DElncRNAs, 19 DEcircRNAs, and 23 DEmiRNAs were predicted by multiple approaches. In general, the DEmiRNAs were selected as the hub components for constructing the ceRNA regulatory network (Table 1). Targeting relationships between DEmiRNAs–DEmRNAs, DEmiRNAs–DElncRNAs, DEmiRNAs–DEcircRNAs were predicted mainly based on miRanda (v1.0b, -sc 100; -en -20). In addition, miRcode (http://mircode.org/), TargetScan (http://www.targetscan.org/), starBase (http://starbase.sysu.edu.cn/) and CircInteractome (https://circinteractome.irp.nia.nih.gov/) were also exploited to confirm the targeting relationships. The overlapping DEmiRNAs predicted in all the three RNA-RNA pairs were then used as core nodes to build the initial ceRNA regulatory network in Cytoscape (v3.8.2). The complete circRNA/lncRNA–miRNA–mRNA regulatory network was finally constructed based on the predicted targeting relationships between miRNAs and other RNAs.

### Quantitative real-time PCR

To evaluate mRNA, lncRNA, and circRNA expression profiles, first strand cDNA was reverse transcribed with oligo (dT) plus random hexamer primers using M-MuLV reverse transcriptase (FAPON, China). Quantitative real-time PCR was conducted on ABI StepOne plus real-time PCR system (Applied Biosystems, USA) with SYBR green master PCR mix and gene-specific primers. Expression levels of targeted genes were normalized by the reference gene (β-actin). For miRNA expression profile evaluation, methods described in our previous study were utilized with snoRNA-202 as reference [44, 47]. Relative expression of all RNAs was calculated according to the 2^−△△Ct^ method. All primers used in this study were listed in Additional file 1: Table S5.

### Diagnostic evaluation of hub DEmRNAs

The mRNA expression dataset of PAH patients (GSE117261) was downloaded from the Gene Expression Omnibus (GEO) database, which includes gene expression profiles of lung tissues from 58 PAH patients (32 patients with idiopathic PAH (IPAH); 5 patients with heritable PAH (HPAH), 17 patients with connective tissue disease, congenital heart defects, anorexigen/stimulant drug use-associated PAH (APAH) and 4 uncharacterized patients) and 25 normal individuals The normalized gene expression pattern of selected genes was analyzed using GraphPad Prism 8.0.1. The diagnostic value of hub DEmRNAs was analyzed by establishing a receiver operating characteristic (ROC) according to their gene expression profile using GraphPad Prism 8.0.1. The AUC value of the ROC curve was calculated for determining the diagnostic effectiveness.

## Supplementary Information

**Additional file 1: Table S1.** RVSP and RVHI in three independent batches batch of HPH rats. **Table S2.** Information of raw reads, clean reads, and reads mapped to rat genome or miRNAs. **Table S3.** Gene Ontology (GO) analysis of differentially expressed mRNAs (DEmRNAs) in the pulmonary arteries of HPH rats. **Table S4.** Kyoto Encyclopedia of Genes and genomes (KEGG) pathway analysis of differentially expressed mRNAs (DEmRNAs) in the pulmonary arteries of HPH rats. **Table S5.** Primers used in this study.

## Data Availability

NGS data have been uploaded to the NCBI Sequence Read Archive database. The data is available in the BioProject PRJNA809145.

## Abbreviations

ceRNA: Competitive endogenous RNA
HPH: Hypoxia-induced pulmonary hypertension
DERNAs: Differentially expressed RNAs
PAH: Pulmonary artery hypertension
GEO: Gene Expression Omnibus
PASMCs: Pulmonary artery smooth muscle cells
IGF1R: Insulin-like growth factor receptor
PPI: Protein–protein interaction
qRT-PCR: Real-time reverse transcription-PCR
RVSP: Right ventricular pressure
RVHI: Right ventricular hypertrophy index
H&E: Hematoxylin and eosin
FPKM: Fragments per kilo base per million mapped reads
TPM: Transcript per million
GO: Gene ontology
KEGG: Kyoto Encyclopedia of genes and genomes
HPAH: Heritable PAH
APAH: Associated PAH
LTBP2: Latent transforming growth factor beta binding protein 2
POSTN: Periostin
SPP1: Secreted phosphoprotein 1
LSAMP: Limbic system associated membrane protein
AUC: Area under the curve
CI: Confidence interval
MREs: MiRNA binding sties
CAMK2D: Calcium/calmodulin-dependent kinase II-delta
CNN3: Calponin 3
MCT: Monocrotaline
iNOS: Inducible nitric oxide synthase
NO: Nitric oxide
RVFbs: Right ventricular fibroblasts
HySU: Hypoxia plus SU5416
ECM: Extracellular matrix
IPF: Idiopathic pulmonary fibrosis
OSCC: Oral squamous cell carcinoma
EMT: Epithelial-mesenchymal transition
SD: Sprague–Dawley
CNCI: Coding-non-coding-index
CPC: Coding potential calculator
PFAM: Pfam Scan
BP: Biological processes
CC: Cellular component
MF: Molecular function
ROC: Receiver operating characteristic
